# Overweight and Obesity in Portuguese Children: Prevalence and Correlates

**DOI:** 10.3390/ijerph111111398

**Published:** 2014-11-03

**Authors:** Thayse Natacha Gomes, Peter T. Katzmarzyk, Fernanda K. dos Santos, Michele Souza, Sara Pereira, José A. R. Maia

**Affiliations:** 1 CIFI^2^D, Kinanthropometry Lab, Faculty of Sport, University of Porto, Rua Dr. Plácido Costa, 91, 4200-450 Porto, Portugal; E-Mails: thayse_natacha@hotmail.com (T.N.G.); fernandak.santos@hotmail.com (F.K.S.); mcsouza85@hotmail.com (M.S.); sara.s.p@hotmail.com (S.P.); 2 Pennington Biomedical Research Center, Louisiana State University, 6400 Perkins Rd., Baton Rouge, LA 70808, USA; E-Mail: Peter.Katzmarzyk@pbrc.edu; 3 CAPES Foundation, Ministry of Education of Brazil, Brasília DF 70040-020, Brazil

**Keywords:** overweight, obesity, children, school, correlates

## Abstract

There are widespread differences in overweight/obesity prevalence in children, and understanding the reasons for this is very important. The present study aims: (I) to conduct a meta-analysis on overweight/obesity prevalence in Portuguese children; (II) to identify differences in biological and behavioural characteristics between normal-weight and overweight/obese children; and (III) to investigate the importance of individual- and school-level correlates of variation in children’s BMI using multilevel modelling. A search was done for all published papers including Portuguese children during the last decade; further, 686 Portuguese children (9–11 years old) were sampled and their BMI, family income, maturity offset, nutritional habits, physical activity, sedentariness, sleep time, and school environment information were collected. Results showed a stabilization of overweight/obesity during the last decade, 30.6% (95%CI: 0.287–0.34) for boys, 28.4% (95%CI: 0.23–0.35) for girls, and 30.3% (95%CI: 0.27–0.34) for boys and girls together. Differences between weight groups were only found in individual-level biological traits. The multilevel analysis did not identify significant contributions of school-level variables to children’s BMI variation. In conclusion, no increase was found in the prevalence of overweight/obesity among Portuguese children since 2000. Normal-weight and overweight/obese children only differ in individual-level characteristics, and school context variables were not related to variation in BMI.

## 1. Introduction

Given the systematic rise in the prevalence of overweight/obesity in youth during the last decades [1,2], determining associated factors in children is necessary, especially to design efficient strategies targeting obesity/overweight reduction. Among Portuguese children, Bingham *et al*. [3] reported that being male, having been breastfed, having been born from mothers who did not smoke during pregnancy, engaging in little sedentary behaviour, performing at least 1 hour of moderate physical activity daily, and having parents with higher education levels and a healthy body mass index (BMI) were protective factors against childhood overweight/obesity. Strategies implemented to reduce overweight/obesity in children, especially at the school level, have reported inconclusive outcomes—some showed significant results [4,5], while others did not [6,7].

It has been suggested that overweight/obese children differ in their behavioural and dietary habits from those with healthy weight [8], namely that they tend to have a higher consumption of fat and a lower carbohydrate intake [9], engage in more sedentary activities (such as watching TV, movies, or playing video games) [10], and spend less time in moderate-to-vigorous daily physical activities [11], although this unhealthy profile is not always observed [12]. For example, Maier *et al.* [13] did not find differences between normal-weight and overweight children in total energy and macronutrient intake. In addition, when studying differences in physical activity and sedentary behaviour among Chinese youth, Wang *et al.* [14] reported no differences among normal-weight, overweight or obese youth.

The prevalence of overweight and obesity among Portuguese children may have increased during the last decades [15] leading to concern among public health authorities [16]. A better understanding of differences between normal-weight and overweight/obese children’s lifestyle characteristics is needed to reduce the negative behavioural and health effects of excessive weight in childhood. Further, as children spend most of their daily time at school, where healthy behaviours are learned [17], the school environment may promote healthy habits that positively affect children’s weight status [18].

In order to better understand the prevalence and correlates of overweight and obesity in Portuguese children, the present study aims to: (I) conduct a meta-analysis on overweight/obesity prevalence in 9 to 11 year old Portuguese children, (II) detect significant differences in behavioural characteristics among normal-weight and overweight/obese children; and (III) investigate the importance of individual- and school-level correlates on variation in children’s BMI.

## 2. Methods

To address this study’s aims, we present the methodology in two parts: Part I addresses the meta-analysis and Part 2 focuses on aims II and III.

### 2.1. Part I: Meta-Analysis of Obesity Prevalence among Portuguese Children

Between June and July 2014, an online search was conducted using *Scopus, Pubmed* and *Scielo* databases to find all available articles reporting overweight and obesity prevalence in Portuguese children using the following keywords: *overweight*, *obese*, *obesity*, *children*, *youth*, *Portugal*, *Portuguese* and their respective translation to Portuguese by the first author. In addition, another search was done at the Faculty of Sport, University of Porto central library, and Portuguese Statistics databases with the same keywords. Valid papers, or Health Directorate Reports, were included if they: (I) were published between January 2000 and May 2014 because *International Obesity Task Force* (IOTF) cut-points were first published in 2000; (II) sampled Portuguese children aged 9 to 11 years [to be in agreement with The *International Study of Childhood Obesity, Lifestyle and the Environment* (ISCOLE) study sample age range, as mentioned below]; (III) used national samples; (IV) reported overweight/obesity prevalence; (V) used BMI to assess overweight/obesity; (VI) used IOTF [19] cut points to define overweight and obesity; and (VII) were published in English or Portuguese.

A meta-analysis of overweight/obesity prevalence was conducted using The Comprehensive Meta-Analysis v2.2.64 software [20]. Prevalence, 95% confidence intervals, Q-test and I^2^ statistic were computed according to algorithms implemented in the software. Further, the software was also used to assess effect size heterogeneity as advocated by Borenstein *et al*. [21] and Beretvas [22], and fixed and random effects models were used.

### 2.2. Part II: Correlates of Childhood Overweight and Obesity

#### 2.2.1. Sample

The sample of the present study is part of ISCOLE, a research project conducted in 12 countries (Australia, Brazil, Canada, China, Colombia, Finland, India, Kenya, Portugal, South Africa, the United Kingdom, and the United States of America) from all major regions of the world. Its main aims are to determine the relationship between behaviours and obesity in a multi-national study of children aged 9 to 11 years, and to investigate the influence of higher-order characteristics such as behavioural settings, and physical, social and policy environments, on the observed relationship within and between countries [23]. Details regarding the ISCOLE study design and methodology were previously reported elsewhere by Katzmarzyk *et al*. [23].

The sample of the present study comprises 777 Portuguese children, aged 9 to 11 years, from 23 schools from the North of Portugal. In each school, after the project was approved by the Physical Education Department, school Principal and Parental Council, all 5th grade students were invited to enrol in ISCOLE, but only those children aged 9 to 11 years old were classified as eligible. From those, approximately 30 to 40 children per school were randomly selected (50% of each sex). Non-response was negligible (response rate was 95.7%), and missing information was at random (differences between subjects with missing information and those included were not statistically significant). The study protocol was approved by the University of Porto ethics committee, as well as by schools’ directorate councils. Written informed consent was obtained from parents or legal guardians of all children.

#### 2.2.2. Anthropometry

Height, sitting height and weight were measured according to standardized ISCOLE procedures and instrumentation [23]. Height was measured using a Seca 213 portable stadiometer (Hamburg, Germany) without shoes, with the head in the Frankfurt Plane, and sitting height was measured while seated on a table with legs hanging freely and arms resting on the thighs. Body mass was determined with a portable Tanita SC-240 scale (Arlington Heights, IL, USA), after all outer clothing, heavy pocket items and shoes were removed. Two measurements were taken on each child, and a third measurement was taken if the difference between the previous two was outside the permissible range for each measure and its replica (0.5 cm for height and sitting height, and 0.5 kg for weight). The mean value of each measured variable (closest two measurements) was used for analysis. BMI was computed using the standard formula [weight(kg)/height(m)^2^], and subjects were classified as normal weight, overweight and obese according to the IOTF cut-off points suggested by Cole *et al*. [19].

#### 2.2.3. Family Data

Information regarding family environmental characteristics was obtained from a questionnaire completed by parents or legal guardians (see ISCOLE Demographic and Family Health Questionnaire in Katzmarzyk *et al*. [23]). The questionnaire collected information on basic demographics, ethnicity, family health and socioeconomic factors. For the present study, we used information regarding familial socioeconomic status (SES) and parental BMI. SES was defined according to annual family income, ranging from <€ 6,000 to ≥€ 42,000, and subjects were classified in two categories (<€23,999; ≥€24,000).

#### 2.2.4. Biological Maturity

Using age, sex, sitting height, stature and body mass, a biological maturity estimate was obtained using the Mirwald *et al*. regression equations [24]. The set of equations, jointly labelled as maturity offset equations, estimates the timing to peak height velocity (PHV) occurrence. A positive (+) offset expresses the number of years a child is beyond PHV; a negative score (–) signifies the number of years a child is from PHV; a value of zero indicates that a child is presently experiencing his/her PHV.

#### 2.2.5. Nutritional and Behavioural Habits

Information on diet and lifestyle was obtained from a questionnaire answered by each child [23], which includes questions about the frequency of consumption of different types of food in a typical week. Information related to fruits, vegetables, sweets, soft drinks and fast food consumption was assessed. Using principal components analysis, dietary scores were derived for each child from the children’s Food Frequency Questionnaire [23] food groups as input variables (excluding fruit juices), expressing children’s dietary patterns. Reported frequencies were converted into portions/week. Eigenvalues and a scree plot analysis were used as the criteria for deciding the number of components extracted. The two criteria led to similar conclusions, and two factors were chosen for each analysis. The components were then rotated with an orthogonal varimax transformation to force non-correlation of the components and to enhance the interpretation. The component scores computed for each subject for both dietary patterns were standardized to ensure normality. The two components were named “unhealthy food” (e.g., hamburgers, soft drink, fired food, *etc*.) and “healthy food” (e.g., vegetables and fruits). Time spent watching TV during the week was reported by children, and then categorized according to screen time recommendations (<2 h/day and ≥2 h/day). Children also reported whether or not if they had a TV available in their bedroom, as well as their main transportation method to/from school.

#### 2.2.6. Physical Activity, Sedentary Time and Sleep

Actigraph GT3X+ accelerometers (ActiGraph, Pensacola, FL, USA) were used to monitor physical activity, sedentary time and sleep. Children wore the accelerometer at their waist on an elasticized belt, placed on the right mid-axillary line 24 h/day, for at least seven days, including two weekend days. To be eligible for this analysis, children had at least four days (including at least one weekend day) with a minimum of 10 h of wear time per day. From the original sample of 777 children, 686 children fulfilled this condition. Accelerometer information was divided into daytime activities and nocturnal sleep time using an automated algorithm [25,26]. Non-wear time during the awake period was defined as any sequence of at least 20 consecutive minutes of zero activity counts [26].

Different activity phenotypes were determined using cut-points developed by Evenson *et al*. [27]. For the present study, mean moderate-to-vigorous physical activity (MVPA) and mean sedentary time were used, which were defined as greater than or equal to 574 activity counts and less than or equal to 25 activity counts using 15 seconds epochs, respectively.

The nocturnal sleep time for each participant was determined using a novel and fully-automated algorithm specifically developed for use in ISCOLE and other epidemiological studies employing a 24-hour waist-worn accelerometer protocol in children [26,28]. Mean sleep time across all days was used in the analyses.

#### 2.2.7. School Environment

Information concerning the school environment was obtained via a questionnaire (ISCOLE School Environment Questionnaire presented in Katzmarzyk *et al*. [23]) completed by the physical education teacher or the school principal. For the present study we primarily considered the following aspects of the school physical activity environment: the percentage of students participating in school sports or PA clubs; school promotion of active transportation (allowing children to bring their bicycles); student access to a gymnasium during school hours and outside school hours; student access to playgrounds during school hours; student access to sports equipment outside of school time; student access to a cafeteria at school; student access to food and drink vending machines; and student access to fast food restaurant close to school.

#### 2.2.8. Statistical Analysis

Differences in means and frequencies of biological and behavioural characteristics between groups were computed using Student-t and χ^2^ tests. SPSS 20.0, and WinPeppi software [29] were used for these analyses. The extraction and identification of dietary patterns were performed in the SAS 9.3 (SAS Institute Inc., Cary, NC, USA, 2011).

To answer aim III and given data dependency, students nested within schools, a multilevel approach was used and the analysis was done in SuperMix software [30] allowing a simultaneous estimation of all model parameters using maximum likelihood procedures. A series of hierarchical nested models were fitted to explain variation in children’s BMI using the Deviance statistic as a measurement of global fit [31]. Additionally, the relevance of predictors to explain variation in BMI was assessed with a pseudo-R^2^ statistic, which is interpreted as a proportional reduction in variance for the parameter estimate resulting from the use of one model as compared to a previous one [31]. Modelling was done in a “stepwise” fashion as generally advocated [32,33]. Firstly, a null model (M_0_) was fitted to the data to compute the intraclass correlation coefficient to estimate the variance accounted for by the school effects in BMI. Secondly, using child-level BMI predictors (sex, biological maturity, mother and father BMI, TV/PC use during weekdays, having a TV in the bedroom, diet categories, time spent in MVPA, sedentariness and sleeping), Model 1 (M_1_) was fitted. Parental BMI, time spent in MVPA, sedentariness and sleeping were centred at the grand mean to facilitate the interpretation of parameter estimates. Thirdly, with the inclusion of school-level predictors, Model 2 (M_2_) was fitted. Statistical significance was set at *p* < 5%.

## 3. Results

### 3.1. Prevalence of Overweight/Obesity among 9–11 Year-Old Portuguese Children

**Figure 1:**
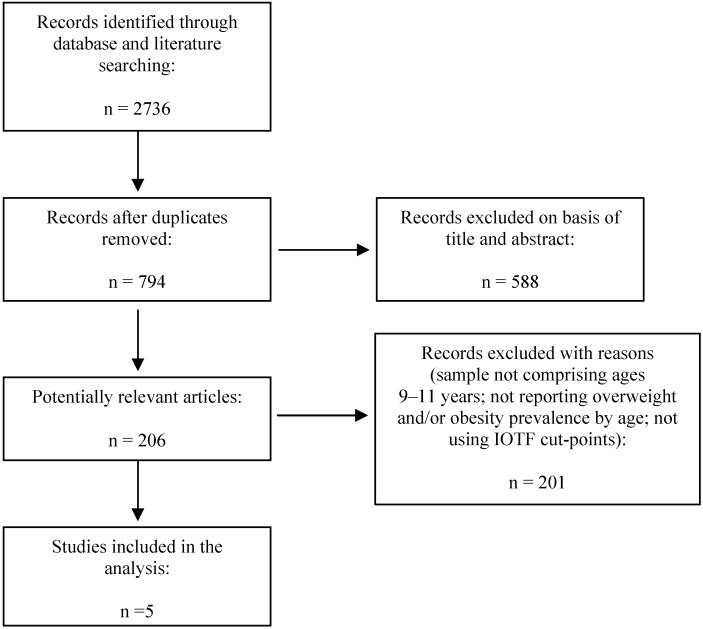
Flow diagram of study selection for meta-analysis.

Figure 1 presents a flow diagram illustrating the search process and the excluded studies in the meta-analysis. Only five studies fulfilled all inclusion criteria based on a close examination of abstracts and full texts. Further, this final list was checked by the first author against two recent systematic literature reviews concerning overweight and obesity in Portuguese children and adolescents [34,35]. Table 1 shows the available data using national samples. Studies were published between 2004 and 2013, and the sample sizes ranged from 405 to 3,584 subjects. The highest prevalence of overweight/obesity was for boys in 2009 [16], while girls in 2007 [36] had the lowest prevalence. A higher prevalence of overweight/obesity was found in boys in three [16,36,37] of the five studies, compared to girls. Taking boys and girls together, the prevalence of overweight/obesity ranged from 19% [37] to 35% [16] representing a moderate-to-high prevalence of Portuguese children with excess weight.

**Table 1:** Summary of overweight/obesity prevalence in 9–11 year-old Portuguese children used in the meta-analysis.

Study	Study Year	Age Range	Sample Size	Prevalence of Overweight/Obesity		
				Boys	Girls	Total
Padez *et al.* [38]	2002/2003	9.5 years	631 (317 boys; 314 girls)	29%	36%	33%
Yngve *et al*. [36]	2003	11 years	1197 (552 boys; 645 girls)	27%	18%	22%
DGS [16]	2008	11 years	405 (204 boys; 201 girls)	39%	31%	35%
Sardinha [37]	2008	10 years	1001 (486 boys; 515 girls)	32%	28%	19%
Bingham *et al*. [3]	2009/2010	9–10 years	3584 (1685 boys; 1899 girls)	31%	32%	31%

**Figure 2:**
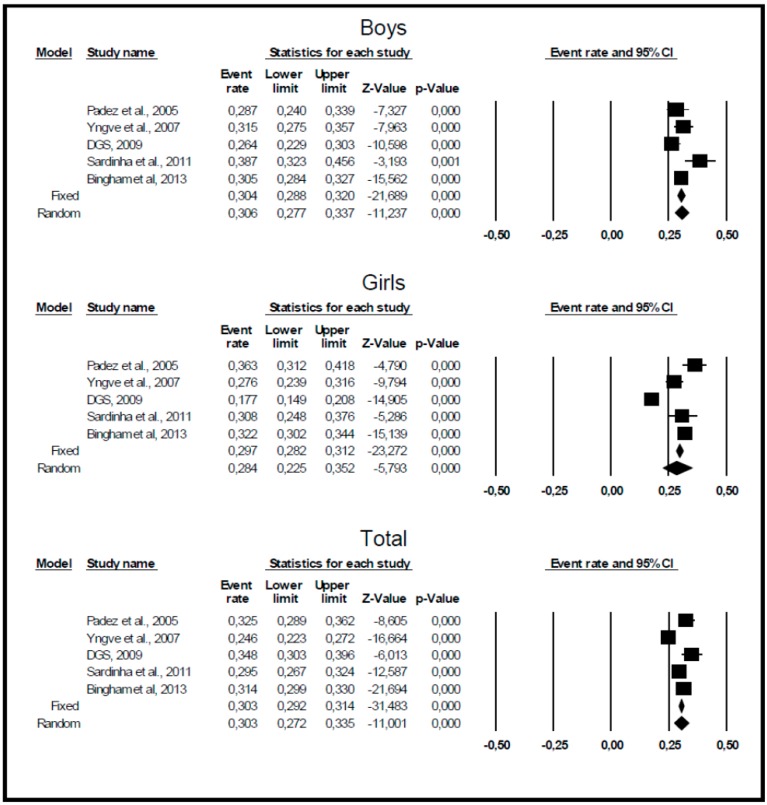
Meta-analysis results for boys, girls and both sexes combined.

Figure 2 presents Forrest plots, fixed and random effects prevalence estimates and their 95% confidence intervals for overweight/obesity across time in boys, girls, and both sexes together. Although there is considerable evidence for heterogeneity in the prevalences for boys (Q-test = 11.371, *p* = 0.023, I^2^ = 64.823), girls (Q-test = 56.564, *p* < 0.001, I^2^ = 92.928), and both sexes together (Q-test = 25.770, *p* < 0.001, I^2^ = 84.478), we nevertheless present fixed and random effects prevalence estimates (see Figure 2) although they are fairly similar. Across the time period, the prevalence estimate for overweight/obesity among boys is 0.306 (95%CI: 0.277–0.337), 0.284 (95%CI: 0.225–0.352) among girls and 0.303 (95%CI: 0.272–0.335) for both sexes. The funnel plot did not show evidence of publication bias; further, meta-regression analysis using study year as a moderator variable did not show any significant increase in overweight/obesity from 2002 till 2010 (boys, beta = 0.0096 ± 0.0132, *p* ≥ 0.05; girls, beta = 0.0220 ± 0.0127, *p* ≥ 0.05; both sexes together, beta = 0.0178 ± 0.0916, *p* ≥ 0.05).

### 3.2. Biological, Behavioural and Socio-Demographic Differences between Normal-Weight and Overweight/Obese Children

Descriptive data comparing differences between normal-weight and overweight/obese children in biological, behavioural and socio-demographic traits are presented in Table 2. Except for biological traits (height, weight, and parental BMI), no statistically significant differences (*p* > 0.05) were found in any of the other variables between the two groups.

**Table 2:** Biological, behavioural and socio-demographic trait differences between normal-weight and overweight/obese children.

	Normal-Weight (n = 417)	Overweight/Obese (n = 269)	*t*	*p-*Value
Height (cm)	141.57 ± 6.39	145.73 ± 6.55	−8.403	<0.001
Weight (kg)	34.14 ± 4.59	47.79 ± 7.74	−28.584	<0.001
Biological maturity	−2.07 ± 0.85	−1.69 ± 0.90	−5.639	<0.001
Unhealthy diet (z-scores)	0.005 ± 1.00	−0.10 ± 0.91	1.424	0.155
Healthy diet (z-scores)	0.04 ± 0.98	−0.03 ± 1.03	0.945	0.345
MVPA (min)	57.34 ± 22.48	55.20 ± 20.54	1.291	0.197
Sedentary time (min)	553.26 ± 61.92	549.68 ± 61.90	0.754	0.451
Sleep time (h)	8.26 ± 0.83	8.26 ± 0.90	-0.027	0.979
**Frequencies (%)**			**χ^2^**	***p-*Value**
SES			0.119	0.730
<€23,999	78.4	79.6		
≥€24,000	21.6	20.4		
Mother BMI			11.744	0.001
Normal-weight	66.3	52.6		
Overweight/obese	33.7	47.4		
Father BMI			4.898	0.027
Normal-weight	41.3	32.2		
Overweight/obese	58.7	67.8		
Transport to/from school			0.339	0.561
Active	28.8	26.8		
Non-active	71.2	73.2		
TV bedroom			3.564	0.059
No	33.1	26.4		
Yes	66.9	73.6		
TV/school day			0.743	0.389
<2 h	73.7	70.7		
≥2 h	26.3	29.3		

### 3.3. Individual- and School-Level Correlates of BMI Variation

Results from M_0_, M_1_ and M_2_ are presented in Table 3. From M_0_, the intraclass correlation coefficient is 0.0216 [0.25/(0.25 + 11.30)], meaning that only 2.2% of the total variance in BMI among schoolchildren is at the school level

Results from M_1_ (child-level predictors) show that boys (*β* = 6.22, *p* < 0.001) have, on average, higher BMI than girls; more mature children had higher BMI (*β* = 3.94, *p* < 0.001); fathers (*β* = 0.12, *p* < 0.001) and mothers (*β* = 0.15, *p* < 0.001) with higher BMIs have children with higher BMIs, suggesting familial aggregation. On the other hand, a higher healthy diet score (*β* = −0.24, *p* = 0.018) showed a negative effect on BMI, as well as MVPA (*β* = −0.02, *p* < 0.001). There was a significant reduction in Deviance from 3621.93 to 2332.03 (χ ^2^ = 1289.90, *p* < 0.001 from M_0_ to M_1_) showing the better fit of this model. Approximately 85% of the variance in BMI was explained by level-1 predictors. The final model, M_2_, which adds school contextual predictors, showed that none were significantly associated with BMI. Although there was a reduction in Deviance from M_1_ to M_2_ (from 2332.03 to 2323.03), it was not significant (χ ^2^ = 9.02, *p* = 0.172). Further, as the intraclass correlation was small (2%), no significant associations in school predictors were expected.

**Table 3:** Multilevel modelling results: regression estimates (*β*), standard-errors (SE), and *p*-values for children and school characteristics influencing BMI variation.

Parameters	Model 0			Model 1			Model 2		
	*β*	*SE*	*p-value*	*Β*	*SE*	*p-value*	*β*	*SE*	*p-value*
Intercept	19.46	0.17	<0.001	23.81	0.41	<0.001	22.77	0.89	<0.001
Sex				6.22	0.35	<0.001	6.23	0.45	<0.001
Biological maturity				3.94	0.20	<0.001	3.96	0.24	<0.001
Mother BMI				0.15	0.02	<0.001	0.15	0.03	<0.001
Father BMI				0.12	0.03	<0.001	0.12	0.03	<0.001
TV weekdays				0.13	0.26	0.622	0.16	0.25	0.540
TV in bedroom				0.34	0.25	0.164	0.32	0.25	0.205
Unhealthy diet				−0.20	0.13	0.142	−0.20	0.12	0.098
Healthy diet				−0.24	0.10	0.018	−0.27	0.12	0.020
MVPA				−0.02	0.00	<0.001	−0.02	0.01	0.002
Sleep time				−0.19	0.12	0.102	−0.18	0.14	0.178
Sports/PA clubs							0.01	0.12	0.966
Incentive for active transportation (bike)							0.56	0.40	0.179
Playground access during school hours							0.24	0.67	0.722
Access to cafeteria							0.64	0.91	0.491
Access to fast food outside school							0.13	0.30	0.680
Access to sports equipment outside school hours							0.11	0.29	0.708
*Model summary*									
Deviance statistic	3621.93			2332.03				2323.03	
Number of estimated parameters	3			13				19	

## 4. Discussion

Since this study was structured according to its three aims: (I) to conduct a meta-analysis on overweight/obesity prevalence in 9 to 11 year old Portuguese children; (II) to detect differences in behavioural characteristics among normal-weight and overweight/obese children, and (III) to investigate the importance of individual- and school-level correlates on children’s BMI variation, the discussion will follow accordingly.

### 4.1. Prevalence of Overweight/Obesity in 9–11 Year-Old Portuguese Children

There is compelling evidence that overweight/obesity in children is one of the most important public health problems worldwide. According to de Onis *et al*. [1], childhood obesity prevalence increased from 4.2% in 1990 to 6.7% in 2010, and it is expected to reach 9.1% in 2020. A recent Portuguese study [15] among 7–9 year-old children, using data from 1970, 1990 and 2002, reported mean increases in BMI during these time periods, with greater increases between 1992 and 2002. However, as the 1970 and 1992 studies did not report overweight/obesity prevalence, it was not possible to determine a trend although they hypothesized that the increase observed from 1992 to 2002 could be linked to increases in the prevalence of overweight/obesity observed in the last years. Our meta-analysis results suggest that the previous increases in obesity may have slowed or plateaued in Portugal. Our findings are in agreement with recent trends of stability or decrease in the prevalence of overweight/obesity in youth. For example, among Dutch, Moroccan and Surinamese South Asian ethnic groups in the Netherlands, aged 3–16 years, de Wilde *et al*. [39] found a stabilization, or even a decrease, in the prevalence of overweight/obesity between 1999-2011. Moreover, similar results were reported by Moss *et al*. [40] studying German children, especially from the year 2004 onward; further, they reported a decline in the prevalence of overweight from 8.4%–11.9% to 3.3%–5.4%, varying according to regions, with an absolute decrease of prevalence rates up to 3% for overweight and 1.8% for obesity. Additionally, among Danish children aged 7–14 years [41] (using self-reported values for height and weight), the prevalence of obesity did not increase significantly from 1995 to 2001 (2.3% to 2.4%), but the prevalence of overweight rose 10.9% to 14.4%). Among French children [42], aged 3–14 years, no significant difference was found in the prevalence of overweight/obesity from 1999 to 2007. Notwithstanding World Health Organization data showing increases in overweight/obesity in young populations [1], it seems that in some nations a levelling off in childhood overweight/obesity has been observed [43,44,45], which calls for a more reliable analysis about variations among countries. In our meta-analysis, the prevalence of overweight/obesity ranged across studies from 19% to 35%, with an overall estimate (from fixed and random effects) of approximately 30% (for boys and girls, together), which is higher than those reported from previous studies. Although one study relied on self-reported height and weight [36], its results were not different from the others.

The reason for the stability in youth overweight/obesity prevalence observed in some countries is not clearly understood, and the same occurs in the present study. Tambalis *et al*. [46] hypothesized that the obesity prevalence may have reached a race and/or country specific ceiling, implying that children with predisposition toward obesity are now obese and obesity prevalence will not increase systematically. Similarly, Olds *et al*. [44] suggested that the environment in developed countries may be saturated with unhealthy food and options for sedentariness that children with a predisposition to becoming overweight have become overweight, and the remaining children may be resilient to obesogenic environments. In the Portuguese context, there is no published evidence to support our results. However, available data on physical activity in this age range (9–11 years), showed that approximately 36% of children achieve the daily recommended levels of MVPA [47]. Additionally, there has been an observed increase in organized sports participation during the last years among Portuguese youth (increases up to 149% from 1996 to 2010) [48] which may also contribute to this stabilization, given the widely reported relationship between overweight/obesity and physical activity.

### 4.2. Biological, Behavioural and Socio-Demographic Differences between Normal-Weight and Overweight/Obese Children

Our results demonstrate that normal-weight and overweight/obese children are significantly different in their biological traits in that overweight/obese children are taller, heavier and ahead in their maturation. This is expected since previous research has shown that early maturing youth usually are taller, heavier and have higher BMI than their later maturing peers [49]. There is also strong evidence showing familial aggregation in BMI, where children with parents with high BMI, tend to have high BMI values [50,51]. For example, among the Chinese Han population, Hu *et al*. [50] reported that children with overweight/obese parents had higher BMI, and Fuentes *et al.* [51], studying Finnish family’s aggregation in BMI showed that when one or both parents were obese, children were more likely to be in the highest quartile of BMI.

Differences between weight groups in nutritional habits, physical activity levels, sedentariness, sleep time and SES were not statistically significant. There is no clear evidence that children with healthy weight differ from those with excess weight in varied sets of behaviours and demographic characteristics [12,13]. For example, with regards to nutritional habits, Yannakoulia *et al.* [12] using a 3-day food record to study food patterns between normal-weight and overweight children, reported no significant differences between groups. Additionally, Garaulet *et al*. [9], investigating the association between energy and nutrient intake with the prevalence of overweight and obesity, found that overweight boys derived a greater percentage of their energy from fat and less from carbohydrates as compared to normal weight boys, whereas overweight girls consumed less carbohydrates than normal-weight ones. Furthermore, Storey *et al*. [52], investigating non-overweight, overweight and obese adolescent diets, showed that non-overweight students consumed significantly more carbohydrate and fiber, significantly less fat and high calorie beverages, and had a higher frequency of consuming breakfast and snacks compared to their overweight or obese peers. Among Portuguese children, no significant differences between weight groups in healthy and unhealthy diet consumption were found, and children from both groups had equal access to healthy and unhealthy foods. One possible explanation for these results may be related to characteristics of Portuguese schools, which offer a nutrient balanced lunch for children. Moreover, they have food policies and controls regarding snacking and fast food, allowing children from both groups to have equal access to healthy and unhealthy food. In addition, schools also have a national program called “education for health” that aims to teach children about healthy choices and healthy living.

Physical activity and sedentary time/behaviour are usually correlated with weight status where normal-weight children are more active and less sedentary [53,54]. However, in our data no differences were found in physical activity levels or time spent in sedentary behaviour. These results are in line with data reported by Wang *et al.* [14] in Chinese children, as well as with those from Maier *et al*. [13]. This last investigation showed that all overweight or obese children reached the recommendation of spending one hour per day in sportive activities which was similar to normal-weight children. In Portugal, physical education is mandatory (twice a week); in addition, all schools offer free sports club participation, and most children (independent of their SES) have access to private sports club which may explain our results.

There is evidence that short sleep duration is consistently associated with concurrent and future obesity [55], where overweight/obese children tend to spend less time sleeping than children of normal weight [56,57,58]. This relationship may be explained by alterations in glucose metabolism, up-regulation of appetite, and decreased energy expenditure [59]. However, our results differ from previous studies in that no differences were found in sleep time between normal-weight and overweight/obese children meaning that sleep deprivation may not be a major risk factor for development of overweight/obesity among Portuguese children. However, our sample included both overweight and obese children, and different results may be obtained by focusing on obesity alone.

In our study SES was determined by annual household income, and it was not found to be different between children of different weight status. Available data do not consistently show a clear effect size and direction in the association between weight status and SES. Previous research suggests that in developed countries, children of low and medium SES are more likely to be obese than those of high SES [60], while others report that higher SES is positively associated with overweight and obesity in Chinese children [61]. Given the distribution of SES in our sample, without any noticeable income inequality, the null results were expected.

### 4.3. Individual- and School-Level Correlates of BMI Variation

The third purpose of the present study was to investigate the importance of individual- and school-level correlates on variation in children’s BMI. At the child level, most variables were significant predictors. Similar to our results, sex differences in BMI have been consistently reported, with boys having higher BMI. For example, Meigen *et al.* [62] studied secular trends in German children’s and adolescents’ BMI and showed a greater increase in boys. Moreover, among Chinese children, Song *et al*. [63] identified sex disparities in BMI-for-age z-score during a 15-year period in which girls were stable whereas a linear increase was observed in boys. Additionally, Ogden *et al*. [64], found a significant increase in obesity prevalence between 1999–2000 and 2009–2010 in American boys as contrasted with girls. Similarly, Skinner and Skelton [65] also reported a stabilization, with a non-significant increase, in the prevalence of obesity among US children from 1999 to 2012. In the Portuguese context, results differ between studies [66], and there is no clear trend for boys having higher BMI than girls.

It is evident that more mature children have higher BMIs [49], as early maturing children are taller and heavier than on time or late maturing peers, where early maturity affects weight relatively more than height [49]. As such, our results were expected since those closer to their PHV (higher maturity offset positive values) had higher BMI values. Further, the relationship between parental and offspring BMI was significant, *i.e.*, children whose parents report high BMI tend also to have high BMI values, which is a consistent finding in previous twin and family studies [50,51]. For example, two Portuguese family studies by Souza *et al*. [67] and de Chaves *et al.* [68] reported the presence of genetic factors explaining from 30% to 50% of the total variance in different body composition phenotypes, which agrees with our findings that parental BMI is positively correlated with their child’s BMI.

Nutritional habits and physical activity levels are two behavioural phenotypes usually associated with BMI [54]. Our results firstly showed that a healthy diet was negatively associated with BMI, meaning that children with a higher healthy diet score had lower BMI values. Among Mexican children [69], food patterns characterized by a high intake of sugary cereals, sweetened beverages, industrial snacks, cakes, whole milk, and sweets were associated with higher risk of overweight/obesity. Moreover, accordingly to Maffeis [70], there is some evidence that obese children show a certain preference for a fatty diet. On the other hand, non-overweight students tend to consume significantly more carbohydrate and fibre, and significantly less fat and high calorie beverages [52]. With regard to physical activity levels, our results are also in line with previous research. For example, Bingham *et al*. [3] found that performing at least 1 hour of moderate physical activity every day is a protective factor against childhood overweight/obesity in Portuguese children. Furthermore, Janssen *et al*. [71], in a review paper examining associations between overweight, dietary and physical activity patterns in youth concluded that increasing physical activity participation was a relevant strategy to prevent and treat overweight and obesity.

School predictors were not significantly associated with children’s BMI. It is possible that the low number of schools (23 schools) and the similarity observed across Portuguese school environments may explain the null results, since only 2% of the BMI variation was attributed to school-level differences. Pallan *et al*. [72] investigated interschool variation in BMI z-scores and also found low intracluster correlations—between 0.9% and 4.2%. Further, they reported that the only school-level variable associated with BMI z-score was time spent in physical education classes (minutes/week).

The present study has several limitations. Firstly, the national studies included in the meta-analysis did not include information from the Autonomous Regions of Madeira and Azores, and this may bias the estimates to an unknown degree. However, one study reported a 29.1% prevalence of overweight/obesity in 11 year-old Azorean children [73], and another [74] showed a prevalence of 31.3% and 25.2% among Azorean 10 year-old girls and boys, respectively, which falls within the range of the confidence interval of the overall prevalence across the decade. Additionally, among 9–11 year-old Madeiran children [75], the prevalence of overweight/obesity ranged from 14.7% to 17.1% for boys and 13.3% to 16.9% for girls, which are lower than the prevalences reported in studies involving mainland samples. Secondly, although we used subjective methods to determine nutritional habits and sedentary behaviour, this is current practice in previous studies cited in the present article. Thirdly, we use self-reported parental height and weight which are suitable proxies for their actual values, and this is usual procedure in epidemiological research [76]. Fourthly, although ISCOLE utilized a validated questionnaire to obtain information concerning TV watching, it is possible that children underestimated their actual TV time. Fifthly, the present study combined overweight and obese children for analyses which may have attenuated effect sizes. Note that our total sample size is 686 children, and the distribution of BMI groups is 60.8% (n = 417) normal-weight, 28.3% (n = 194) overweight, and 10.9% (n = 75) obese. Separate analyses were not performed due to the lack of statistical power; further, our parameter estimates would be less precise, and our conclusions less reliable. Notwithstanding these limitations, the study has several merits: (1) the presentation of a meta-analysis of the prevalence of overweight/obesity in the last decade in Portuguese children within an important developmental transition period; (2) the use of an objective method to assess physical activity; (3) inclusion of objective information regarding sleep time; (4) use of standard methods and highly reliable data; and (5) the use of multilevel modelling to capture the complexity of nested information at children and school levels.

## 5. Conclusions

In summary, the present study showed that in the last decade, overweight/obesity among 10 year old Portuguese children was stable. Normal-weight and overweight/obese children differed in their biological traits, but not in behavioural or sociodemographic traits. School characteristics did not seem to play important roles in BMI variation because they explain approximately 2% of the total variance. On the other hand, child-level variables are important because they explain 85% of the total variance attributable to variables at the subject level. Taken together, this information should be carefully considered by families, school authorities and teaching staff, paediatricians, and planners of intervention studies when designing more efficient strategies to combat the obesity epidemic.
